# Supplementary material for: Prolonged Cold Exposure Negatively Impacts Atlantic Salmon (Salmo salar) Liver Metabolism and Function
Source: Biology (Basel). 2024 Jul 3;13(7):494. doi: 10.3390/biology13070494 (PMC11273521; doi:10.3390/biology13070494)
Supplement: Supplementary file 1 [file biology-13-00494-s001.zip › Supplementary figures_ captions.pdf]

Supplementary Figure S1. Correlation of the ten top factors in dimension 1 (A) and dimension 2 (B) of the PCoA in asymptomatic fish and fish with 'early' and 'advanced' symptoms of liver disease. Positive correlations with each dimension are in red (+1), and negative correlations are in blue (-1), the intensity of the color is in accordance with the correlation coefficient of the factor with the dimension.

Supplementary Figure S2. Hepatic transcript expression levels ($Log_{10}RQ$) of target genes tested in pools (n=9 fish per condition) in asymptomatic (white bars) fish and fish with 'early' (light-grey bars) and 'advanced' (dark-grey bars) symptoms of liver disease. Genes are grouped by function. *Fatty acid synthesis*: **A**) Acetyl Co A carboxylase 1a (*acc1a*), **B**) Acetyl CoA carboxylase 1b (*acc1b*), **C**) Acetyl CoA carboxylase 2a (*acc2a*), **D**) Acetyl CoA carboxylase 2b (*acc2b*), **E**) Fatty acid synthase a (*fasa*), **F**) Fatty acid synthase b (*fasb*); *Fatty acid desaturation*: **G**) Delta 9 acyl-CoA desaturase a (*scda*), **H)** Delta 9 acyl-CoA desaturase b (*scdb*), **I)** Delta 5 fatty acyl desaturase (*fadsd5*), **J)** Delta 6 fatty acyl desaturase a (*fadsd6a*), **K)** Delta 6 fatty acyl desaturase b (*fadsd6b*). *Fatty acid elongation*: **L**) Elongation of very long chain FA protein 2 (*elovl2*), **M**) Elongation of very long chain FA protein 5 a (*elovl5a*), **N**) Elongation of very long chain FA protein 5 b (*elovl5b*); *Sterol synthesis*: **O**) Sterol regulatory element binding protein 1 (*srebp1*), **P**) Sterol regulatory element binding protein 2 (*srebp2*), **Q**) HMG-CoA reductase a (*hmgcra*), **R**) HMG-CoA reductase b (*hmgcrb*); *Lipid transport*: **S**) Fatty acid binding protein 3a (*fabp3a*), **T**) Fatty acid binding protein 3b (*fabp3b*), **U**) Fatty acid binding protein 10a (*fabp10a*), **V**) Fatty acid binding protein 10b (*fabp10b*), **W**) Fatty acid translocase-like (*cd36c*), **X**) Apolipoprotein Eb a (*apoeba*). * Indicates the transcripts that were assessed in individual samples.

Supplementary Figure S3. Hepatic transcript expression levels ($Log_{10}RQ$) of target genes tested in pools (n=9 fish per condition) in asymptomatic (white bars) fish and fish with 'early' (light-grey bars) and 'advanced' (dark-grey bars) symptoms of liver disease. Genes are grouped by function. *Lipid transport*: **A**) Apolipoprotein Eb b (*apoebb*); *Lipid oxidation*: **B**) Acyl CoA oxidase 1 (*acox1*), **C**) Acyl CoA oxidase 3 (*acox3*), **D**) Carnitine palmitoyltransferase 1.1 (*cpt1.1*), **E**) Carnitine palmitoyltransferase 1.2 (*cpt1.2*), **F**) Carnitine palmitoyltransferase 1.3 (*cpt1.3*), **G**) Carnitine palmitoyltransferase 1.4 (*cpt1.4*), **H**) Cholesterol 7-alpha hydroxylase a (*cyp7a1b*); *Transcription factor*: **I**) Peroxisome proliferator activated receptor alpha a (*pparaa*), **J**) Peroxisome proliferator activated receptor alpha b (*pparab*), **K**) Peroxisome proliferator-activated receptor beta 1a (*pparb1a*), **L**) Peroxisome proliferator-activated receptor beta 1b (*pparb1b*), **M**) Peroxisome proliferator-activated receptor beta 2a (*pparb2a*), **N**) Peroxisome proliferator-activated receptor beta 2b (*pparb2b*), **O**) Peroxisome proliferator activated receptor gamma a (*pparga*), **P**) Peroxisome proliferator activated receptor gamma b (*ppargb*), **Q**) Liver x receptor a (*lxra*), **R**) MLX interacting protein a (*mlxipa*), **S**) Carbohydrate response element binding protein a (*cherbpa*), **T**) CCAAT/enhancer-binding protein a (*cebpa*), **U)** CCAAT/enhancer-binding protein b (*cebpb*); *Adipocyte homeostasis*: **V**) Thioredoxin b (*tnxb*); *Inflammation*: **W**) Arachidonate 5-Lipoxygenase a (*5loxa*), **X**) Arachidonate 5-Lipoxygenase b (*5loxb*). * Indicates the transcripts that were assessed in individual samples.

Supplementary Figure S4. Hepatic transcript expression levels ($Log_{10}RQ$) of target genes tested in pools (n=9 fish per condition) in asymptomatic (white bars) fish and fish with 'early' (light-grey bars) and 'advanced' (dark-grey bars) symptoms of liver disease. Genes are grouped by function. *Inflammation*: **A**) Arachidonate 15-Lipoxygenase b-like (*alox15*), **B**) Sirtuin 1 a (*sirt1a*), **C**) Sirtuin 1 b (*sirt1b*), **D**) Activating transcription factor 6 (*atf6*), **E**) Prostaglandin D synthase (*pgds*), **F**) Tumour necrosis factor alpha a (*tnfa*), **G**) Rapamycin target (*mtor*); *Immunity*: **H**) Sacsin Molecular Chaperone (*sacs*), **I**) Immunoglobulin mu heavy chain a (*igma*), **J)** Immunoglobulin mu heavy chain b (*igmb*), **K**) Hepcidin antimicrobial peptide a (*hampa*), **L**) Serum amyloid A5 (*saa5*), **M**) Leukocyte cell derived chemotaxin 2 (*lect2*); *Antioxidant capacity*: **N**) Peroxiredoxin 1 (*prx1*), **O**) Peroxiredoxin 6 (*prx6*), **P**) Catalase a (*cata*), **Q**) Catalase b (*catb*), **R**) Catalase c (*catc*), **S**) Superoxide dismutase putative a (*soda*), **T**) Superoxide dismutase putative b (*sodb*), **U**) Glutathione s-transferase a (*gsta*); *Apoptosis*: **A**) Caspase 3 a (*casp3a*), **B)** Caspase 3 b (*casp3b*), **C)** Caspase 8 (*casp8*). * Indicates the transcripts that were assessed in individual samples.

Supplementary Figure S5. Hepatic transcript expression levels ($Log_{10}RQ$) of target genes tested in pools (n=9 fish per condition) in asymptomatic (white bars) fish and fish with 'early' (light-grey bars) and 'advanced' (dark-grey bars) symptoms of liver disease. Genes are grouped by function. *Growth*: **A**) Growth hormone receptor 1 (*ghr1*), **B**) Growth hormone receptor 2 (*ghr2*), **C**) Insulin growth factor 1 (*igf1*), **D)** Insulin growth factor 2 (*igf2*). * Indicates the transcripts that were assessed in individual samples.
