# Supplementary figures and images for: Prolonged Cold Exposure Negatively Impacts Atlantic Salmon (Salmo salar) Liver Metabolism and Function
Source: Biology (Basel). 2024 Jul 3;13(7):494. doi: 10.3390/biology13070494 (PMC11273521; doi:10.3390/biology13070494)

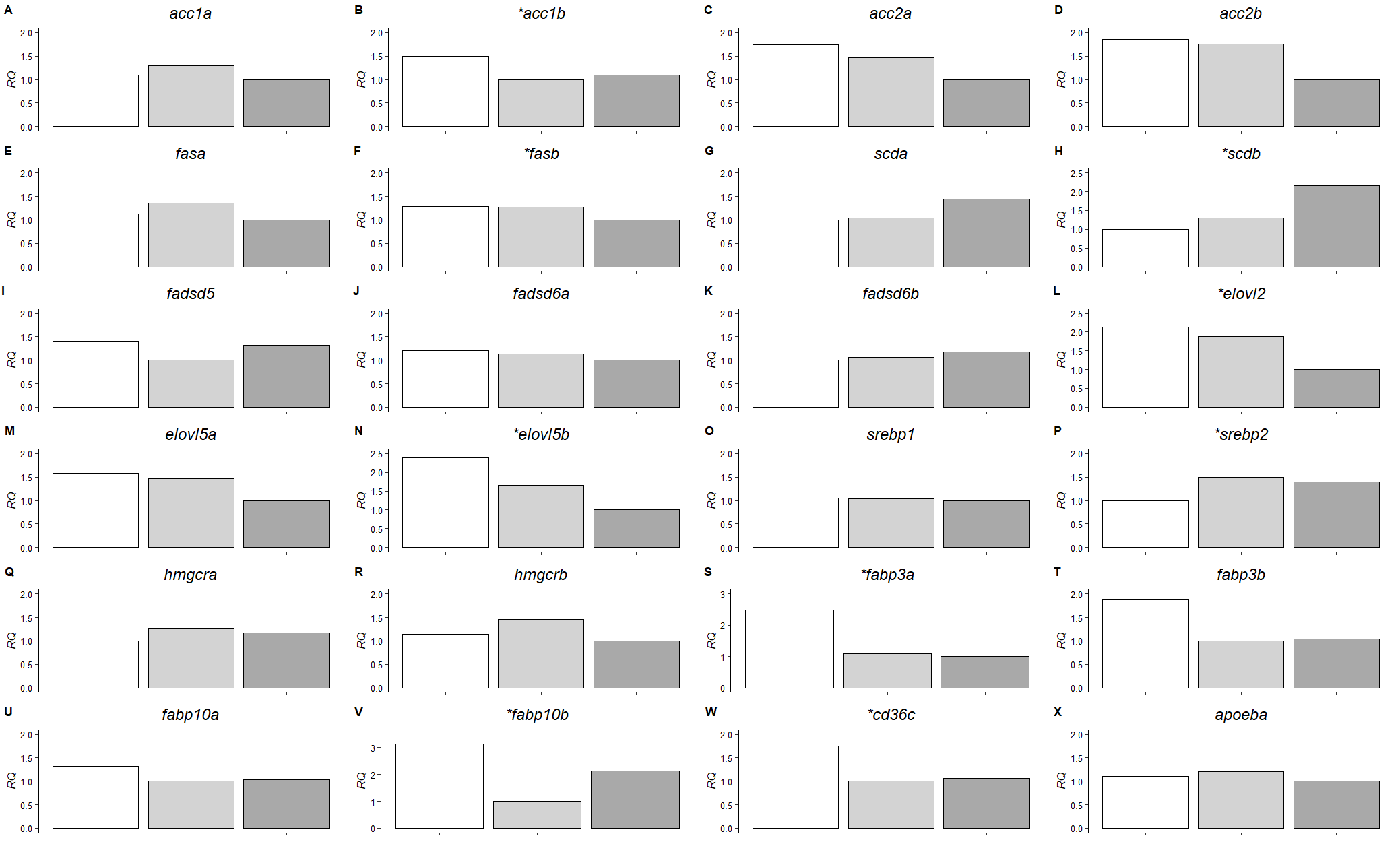

Supplement: Supplementary file 1 [file biology-13-00494-s001.zip › s2.png]

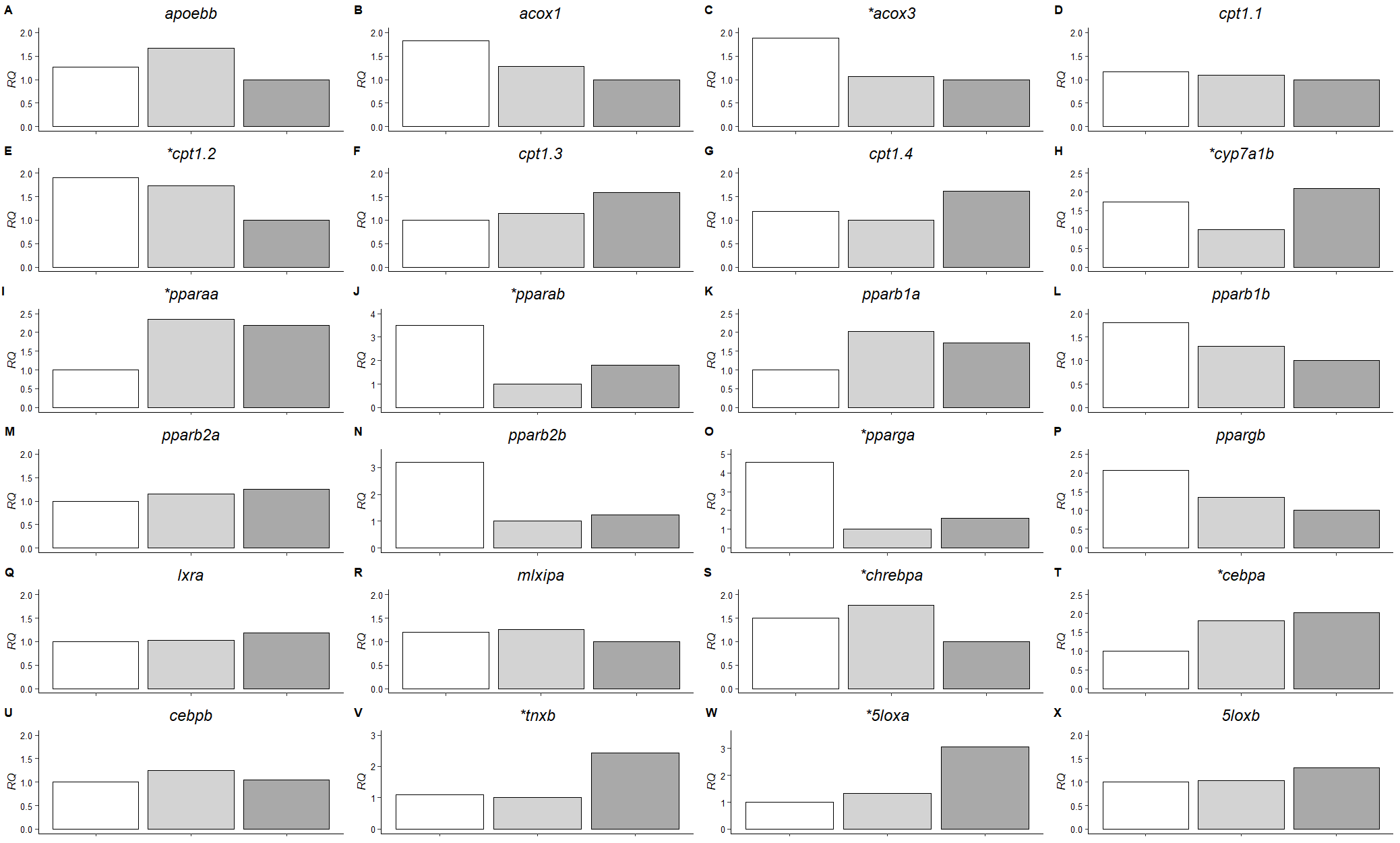

Supplement: Supplementary file 1 [file biology-13-00494-s001.zip › s3.png]

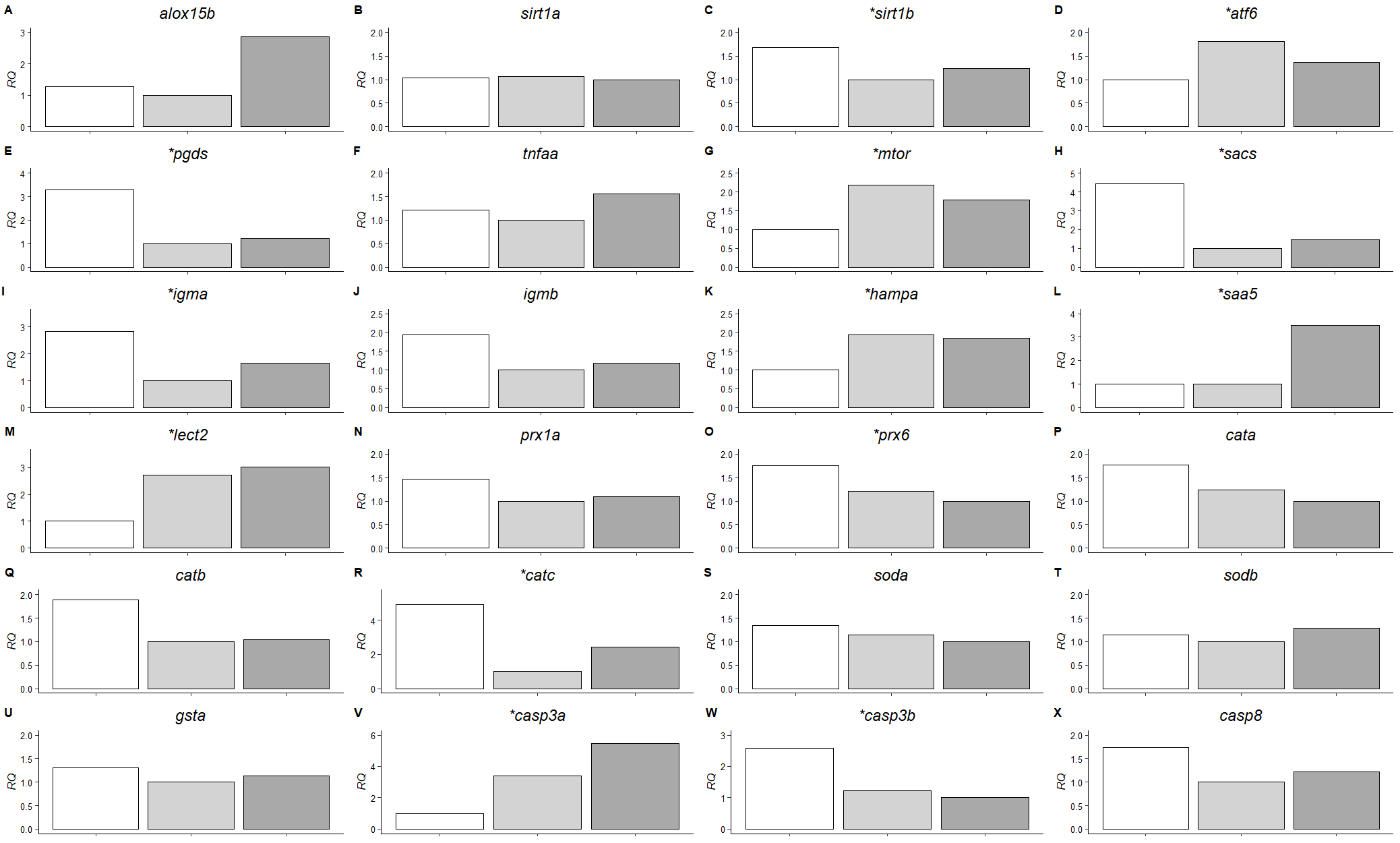

Supplement: Supplementary file 1 [file biology-13-00494-s001.zip › s4.png]

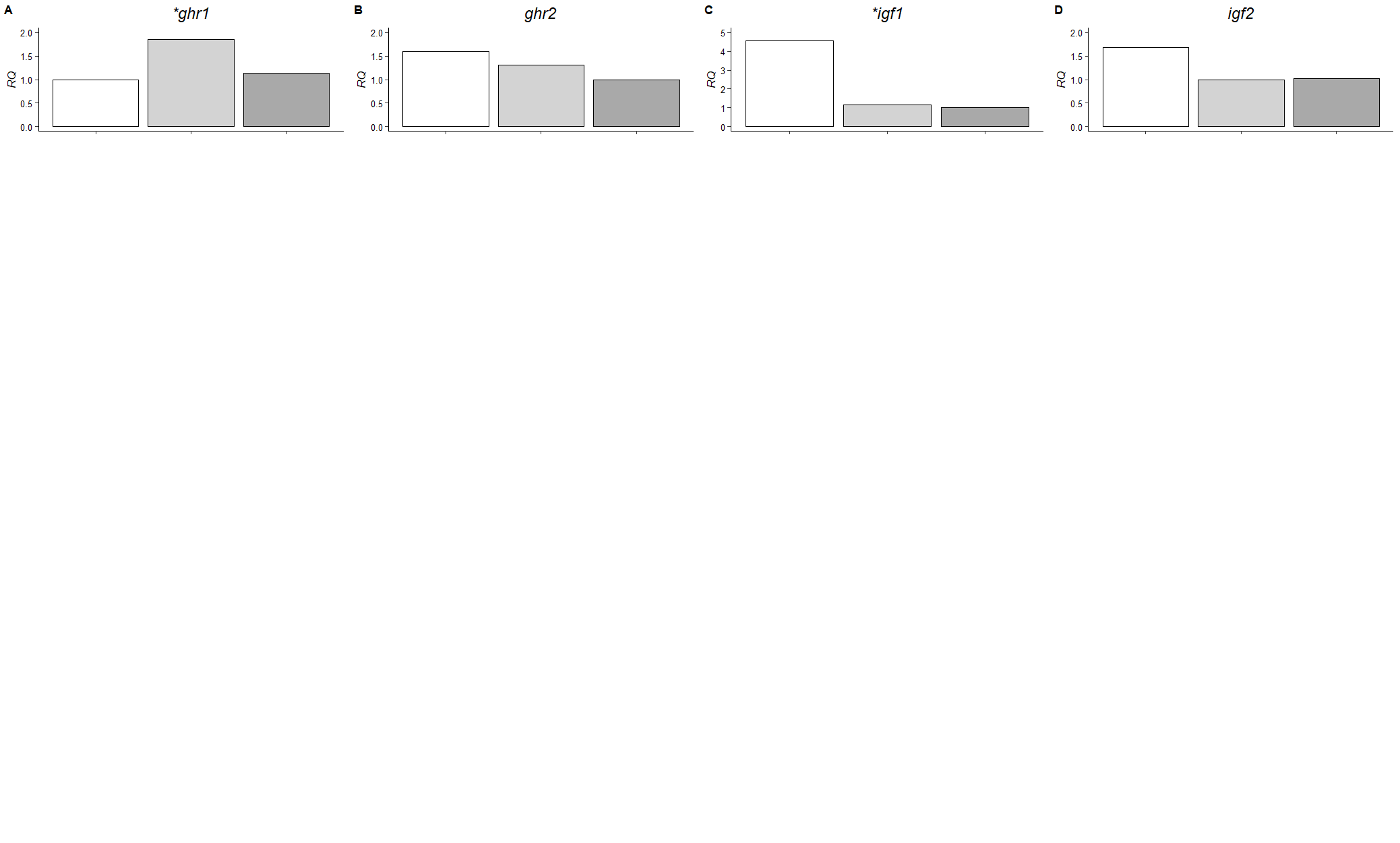

Supplement: Supplementary file 1 [file biology-13-00494-s001.zip › s5.png]
